# Injuries in Runners; A Systematic Review on Risk Factors and Sex Differences

**DOI:** 10.1371/journal.pone.0114937

**Published:** 2015-02-23

**Authors:** Maarten P. van der Worp, Dominique S. M. ten Haaf, Robert van Cingel, Anton de Wijer, Maria W. G. Nijhuis-van der Sanden, J. Bart Staal

**Affiliations:** 1 Academic Institute, University of Applied Sciences Utrecht, Department of Physical Therapy, Utrecht, the Netherlands; 2 HAN, University of Applied Sciences Nijmegen, Institute Health Studies, Nijmegen, the Netherlands; 3 Radboud University Medical Center, Radboud Institute for Health Science, Scientific Institute for Quality of Healthcare, Nijmegen, the Netherlands; 4 Sport Medical Center Papendal, Arnhem, the Netherlands; 5 Radboud University Medical Center, Radboud Institute for Health Science, Department of Oral Function & Prosthetic Dentistry, Nijmegen, the Netherlands; 6 Radboud University Medical Center, Radboud Institute for Health Science, Department of Rehabilitation, Nijmegen, the Netherlands; Delft University of Technology (TUDelft), NETHERLANDS

## Abstract

**Background:**

The popularity of running continues to increase, which means that the incidence of running-related injuries will probably also continue to increase. Little is known about risk factors for running injuries and whether they are sex-specific.

**Objectives:**

The aim of this study was to review information about risk factors and sex-specific differences for running-induced injuries in adults.

**Search Strategy:**

The databases PubMed, EMBASE, CINAHL and Psych-INFO were searched for relevant articles.

**Selection Criteria:**

Longitudinal cohort studies with a minimal follow-up of 1 month that investigated the association between risk factors (personal factors, running/training factors and/or health and lifestyle factors) and the occurrence of lower limb injuries in runners were included.

**Data Collection and Analysis:**

Two reviewers’ independently selected relevant articles from those identified by the systematic search and assessed the risk of bias of the included studies. The strength of the evidence was determined using a best-evidence rating system. Sex differences in risk were determined by calculating the sex ratio for risk factors (the risk factor for women divided by the risk factor for men).

**Main Results:**

Of 400 articles retrieved, 15 longitudinal studies were included, of which 11 were considered high-quality studies and 4 moderate-quality studies. Overall, women were at lower risk than men for sustaining running-related injuries. Strong and moderate evidence was found that a history of previous injury and of having used orthotics/inserts was associated with an increased risk of running injuries. Age, previous sports activity, running on a concrete surface, participating in a marathon, weekly running distance (30–39 miles) and wearing running shoes for 4 to 6 months were associated with a greater risk of injury in women than in men. A history of previous injuries, having a running experience of 0–2 years, restarting running, weekly running distance (20–29 miles) and having a running distance of more than 40 miles per week were associated with a greater risk of running-related injury in men than in women.

**Conclusions:**

Previous injury and use of orthotic/inserts are risk factors for running injuries. There appeared to be differences in the risk profile of men and women, but as few studies presented results for men and women separately, the results should be interpreted with caution. Further research should attempt to minimize methodological bias by paying attention to recall bias for running injuries, follow-up time, and the participation rate of the identified target group.

## Introduction

Although running has been popular since the 1970s [1], the number of runners and running events has increased steadily since 2000 [1,2]. This increase is largely due to girls and women who started running [2,3]. Running in the adult population is one of the most popular physical activities around the world and in the Western society many cities have their own recreational running event. Furthermore, running is one of the most efficient ways to achieve physical fitness, which is linked with longevity [1]. A drawback of the sport is the relatively high risk of injury, with an incidence varying between 19% and 79% [4]. This large variation is due to differences in the definition of injury, study populations, and follow-up periods [5]. Injuries diminish pleasure in exercise and lead to a temporary or even permanent discontinuation of running. Injuries furthermore lead to increased costs because of necessary medical treatment (e.g., the direct medical costs per injured runner at the emergency department is estimated at €1300 [6]), and/or absence from work. In conclusion, running is very popular in the adult population, however strategies are needed to prevent high incidences of running injuries in this group of runners.

Acute running injuries are rare, consisting mainly of muscle injuries, sprain, or skin lesions (blisters and abrasions) [7]. Eighty percent of running disorders are overuse injuries, resulting from a mismatch between the resilience of the connective and supporting tissue and running [7]. Running is one of the most common sports that give rise to overuse injuries of lower back and the leg [8]. The predominant site of leg injuries is the knee, for which the location specific incidence ranged from 7.2% to 50.0% [4]. Running injuries of the lower leg, foot and upper leg are common, ranging from 9.0% to 32.2%, 5.7% to 39.3%, and 3.4% to 38.1%, respectively [4]. Less common sites of running are the ankle, the hip/pelvis/groin and lower back, ranging from 3.9% to 16.6%, 3.3% to 11.5% and 5.3% to 19.1 respectively [4,9–11].

Poorly perfused tissues, such as ligaments, tendons and cartilage, are particularly at risk because they adapt more slowly than muscles to increased mechanical load [7]. Hreljac [8] suggested that injury should be avoided not by minimizing the stress applied to a biological structure but by optimizing the amount and frequency of loading stress. Given the dynamic nature of the relationship between applied stress and injury, there must be an optimal level of applied stress for any biological structure [8].

Furthermore, the multifactorial model of Meeuwisse et al. showed the importance of identifying predisposing factors that make a runner susceptible for injury [12]. Identifying such factors may contribute to the development of injury prevention strategies [13], especially when these can be influenced by adequate training or by optimizing training environment. Moreover, the exact causes of running injuries are likely to be diverse [4] and possibly interacting with each other [13].

Risk factors for running injuries can be clustered into three domains, 1) personal factors (e.g. age, sex, height, genetic imprinting), 2) running/training factors (e.g. weekly running days, distance, running shoes), and 3) health and lifestyle related factors (e.g. smoking, a history of comorbidity and previous injuries) [4]. Three narrative reviews [5,14,15], published in 1992, reported the occurrence of injuries to be based on multifactorial risk factors. In their systematic review, Van Gent et al. (2007) [4] found limited evidence that older age [16], differences in lower leg length [17], a larger left tubercle-sulcus angle [17] and greater knee varus [17], greater height (in men) [18], use of alcohol [16], and a positive medical history (e.g. taken medication, high blood pressure, asthma, and nervous or emotional problems) [16] are associated with a higher risk of injury in men and women. Strong evidence was found that previous injuries were associated with lower extremity running injuries [4], but the studies used different definitions of previous injury, in terms of its location, time of occurrence, etc. Also, the recent systematic review of Saragiotto et al. [19] confirmed that previous injuries are a risk factor for new running injuries and no association between sex and running injuries was found in most of the included studies. In this systematic review [19] only prospective cohort studies were included and risk factors for general running-related injuries were determined. However, no distinction was made in the risk factors for specific running related injuries, e.g. medial tibial stress syndrome, Iliotibial band syndrome, etc.

Differences in the health status of women and men are of increasing concern to European health policymakers and are becoming a subject of growing interest to researchers [20].

The injury patterns between men and women differ and there are several reasons for the differences in injury rates, related to anatomic and physiologic differences [21].

Two recent Dutch prospective studies of novice runners [10,22] pinpointed at possible differences in injury risk profiles of men and women. In a study of runners (n = 629) who were preparing for a 6.7-km run, a younger age and lack of running experience were significant risk factors for running injuries in men, whereas lack of running experience, a higher body mass index (BMI), and earlier participation in sports without axial pressure (swimming and cycling) were risk factors for running injuries in women [10]. A subsequent study of a different cohort of novice runners (n = 532) also showed sex-specific risk factors, but the results were contradictory: significant risk factors for men were previous injuries in the past year, higher BMI, and earlier participation in sports without axial pressure, whereas in women a positive navicular drop test was the sole risk factor in adjusted analyses [22]. However, the statistical analysis used in these two studies, stepwise multiple regression, is questionable [23] and more research is needed to clarify the sex difference in risk profile.

A previous study from Canada also reported sex differences in risk factors for running injuries. A BMI of > 26 kg/m2 was reported as protective in men, whereas age younger than 31 years was protective in women; running once a week and age older than 50 years were risk factors in women [24].

From the above, it can be appreciated that it is difficult to draw conclusions about the risk factors for running injuries in general, for specific running injuries, and possible differences in risk profile between men and women. Earlier reviews [4,5,14,15] need to be updated to identify all possible factors that may predispose a runner for injury and enabling future researchers to develop, potentially sex-specific, interventions to prevent running-related injuries [13].

The present literature synthesis aims to review current evidence for risk factors for running-associated injuries in adults and to determine whether risk factors for such injuries differ between men and women.

## Methods

We used the MOOSE statement to report our systematic review of observational studies and the STARLITE statement to report our literature search [25,26].

### Search Strategy

Four bibliographical databases, namely, CINAHL (1982 to 26 December, 2012), EMBASE (1947 to 1 January, 2013), PubMed (1940 to 26 December, 2012), and Psych INFO (1806–1 January, 2013), were searched using search strings developed by the first author and the librarian expert (AT) of the Radboud University Nijmegen Medical Center. The following search terms (Mesh, title- and/or abstract words) were used to identify the study population in combination with lower extremity injuries: running, track and field, jogging and lower limb, lower extremity, leg-, hip-, knee-, ankle- and foot injuries, soft tissue injuries, musculoskeletal pain, bursitis, sprains and strains, tendinopathy, tendinitis, Iliotibial band syndrome, patellofemoral pain syndrome, and plantar fasciitis. Keywords used to identify a relevant study design were cohort studies, longitudinal studies, follow-up, retrospective-, observational-, prospective studies, risk factors, and etiology. For the PubMed search, see S1 Appendix. The search strings of the other databases are available upon request from the authors.

### Selection Criteria

Two reviewers (MvdW & JS) independently selected relevant articles, based on titles and abstracts. Full papers were retrieved if the abstract provided insufficient information to decide whether the article should be included. The selection criteria were: 1) the design indicated a longitudinal cohort study with a minimal follow-up of 1 month; 2) the objective of the study was to investigate the association between risk factors (personal factors, running/training factors and/or health and lifestyle factors) and the occurrence of lower limb injuries; 3) the study population consisted of novice runners, long-distance runners both recreational and/or competitive; 4) the article was published in a peer-reviewed journal in English or German. Studies concerning elite, professional or ultra-runners, patient populations, children, and/or young adolescents (age <18 years), or in which participants were predominantly exposed to other types of sporting activity than running (e.g. military training, triathlon, etc.) were excluded. If a study contained a mixed population of runners and patients, the results for the runners had to be presented separately in order for the study to be included. The reference lists of all identified relevant publications were checked for other relevant publications.

### Quality Assessment

Articles that met the selection criteria were evaluated for risk of bias. A quality list of twelve items, based on assessment tools of the Cochrane Collaboration [27] and previous systematic reviews of risk factors for musculoskeletal disorders [28–30], was used. The list was based on generally accepted principles of etiological research and was relevant for cohort studies. Some items were adapted to the topic of interest of this review by replacing risk factors with personal factors, running/training factors, and/or health & lifestyle factors (see S1 Table).

Two reviewers (DtH & MvdW) independently assessed the quality of the studies. All items were scored as positive, negative, or unclear. A positive score indicated a well-described and well-performed item. A negative score indicated that the item was described but not well performed, and unclear meant the item was unclear because insufficient information was available. For each item, the scores of the two reviewers were compared. Any difference in scoring was resolved in a consensus meeting. If consensus was not reached, a third reviewer (AW) made the final decision. A high-quality study was defined as scoring positive on > 50% of the items [28–30].

### Data Extraction and Statistical Analysis

The following information was extracted from the included studies: year of publication, study design with follow-up period, injury definition, population characteristics (age, sex, body mass index, or height and weight, and the proportion of subjects analyzed in the included studies; number of subjects analyzed, divided by the number of included subjects, multiplied with 100) and the incidence of (running) injuries; injury specific or overall and, if given, sex specific.

Cohen’s Kappa (K) values were calculated for the interobserver agreement between the two reviewers with regard to risk of bias. A Kappa value of > 0.8 indicates high level of agreement between assessors, a value between 0.61 and 0.8 a substantial agreement, a value between 0.41 and 0.6 a moderate level of agreement, and a value of < 0.41 poor level of agreement [31]. SPSS 20.0 was used to calculate K values.

The main dependent outcome variable was running-induced leg injury. Identified risk factors were summarized per injury, overall and injury specific. All risk factors were grouped into three main categories: 1) personal factors, 2) running/training related factors, and 3) health & lifestyle related factors.

To evaluate associations between risk factors and running injuries p-values, crude odds ratios (ORs), hazard ratios (HRs,) and relative risks (RRs) with 95% confidence intervals (CI) were retrieved from the included publications. Crude values were used for this evaluation to prevent biases and shortcomings of stepwise multiple regression analyses [23]. Adjusted risk estimates derived from multivariable regression analyses were only used when the independent variables of the model were pre-specified and not based on a stepwise selection algorithm or when crude associations were not available.

### Pooling and Best-Evidence Synthesis

Separate meta-analyses with the random effects model [32] were planned to obtain the pooled OR, HR or pooled RR (with 95% CI) for running injuries. If pooling was not possible due to heterogeneity of the study populations, a best evidence synthesis was presented.

For each identified risk factor, levels of evidence were established for the association between this factor and the occurrence of running injuries. These levels of evidence were based on the guidelines of van Tulder et al. [33] and were divided into the following levels: strong evidence, defined as consistent findings (in ≥75% of the studies) in multiple (≥ 2) high-quality studies; moderate evidence, defined as consistent findings (in ≥75% of the studies) in one high-quality study and multiple low-quality studies; limited evidence, defined as consistent findings (in ≥75% of the studies) in multiple low-quality studies or one high-quality study; and conflicting evidence, defined as conflicting findings reported by <75% of the studies reporting consistent findings.

### Sex Ratio

In studies in which risk factors were presented separately for men and women, possible sex differences in risk were determined by dividing the risk factor for women by the risk factor for men, which produced a sex ratio. A ratio higher than 1.25 (i.e., women had a higher risk) or lower than 0.75 (i.e. women had a lower risk) was regarded as a relevant sex difference [34,35].

## Results

### Literature Search

A flow chart for article retrieval is given in S1 Fig. Of 400 articles retrieved as potentially relevant, 17 were considered eligible for full-text screening based on title and abstract. Of these 17 studies, 2 [36,37] seemed, after consultation with the authors, to have an abstract only, so 15 articles were included for quality assessment, data extraction, and analysis.

### Included Studies

Of the 15 included longitudinal cohort studies, 13 were prospective [10,17,22,24,38–46] and 2 retrospective [9,47] studies. They were all published in English. The follow-up time of these studies ranged from 8 weeks to 1 year and the mean age of study participants ranged from 36 to 44 years. Thirteen studies had a mixed population, 1 study included only women [39], 1 study included only men [42], and 1 study did not report the sex of the study population [47]. BMI and height differed between the various reports. In the study of Bennett et al. [38], 13.6% of study participants had a low BMI (<18.5 kg/m2); the studies of Lun et al. [44] and McKean et al. [47] did not report BMI, weight, or height. The proportion of subject analyzed in the original studies ranged from 46% to 100%. Seven studies [10,22,24,39–41,43] included novice runners. All studies used different (running) definitions of injury, except for one research group who used the same definition in their two studies [9,10,17,22]. Five studies defined running-related injuries as involving the lower limb [38,40,41,43,46], 4 studies included the influence of the symptoms on running [9,17,24,47], and 6 studies defined injuries in terms of the lower limb and the influence of symptoms [10,22,39,42,44,45]. Two studies included a specific time frame of running restriction caused by the running injury [10,22]. Four studies specifically looked at signs and symptoms related to Achilles tendinopathy [41,46] and patellofemoral dysfunction/pain syndrome [39,43]. Only Bennett et al. [38] excluded traumatic injuries. Wen et al. [9,17] included overuse injuries in their definition of injury. Wen et al. [9,17] investigated the same experienced runners in a retrospective study [9] and in a longitudinal prospective study, published a year later [17]. In order to avoid duplication, the results of these two studies were considered as coming from one study population [48]. The incidence of the running injuries reported in the included studies where in the range of 20.6% to 79.3%, 25.0% to 79.5% and 19.8% to 79.1% for overall, men and women, respectively. The injury specific incidences were 7.8% and 14.3% for Achilles tendinopathy injuries [41,46] and 16.7% and 20.8% for patellofemoral pain injuries [39,43]. S2 Table presents a summary of these studies including the population characteristics (age, sex, BMI, and the proportion of people analyzed), type of running, injury definition and (running) incidence; injury specific and/or overall and, if given, sex specific.

### Risk of Bias

The overall agreement between the two reviewers was 77% with a moderate reliability (Kappa = 0.6). The agreement for the individual items ranged from 53% (item 12) to 100% (item 6). Most disagreement was seen for item 5 (“Were the data on system factors, running/training related factors, and/or health and lifestyle factors collected using standardized methods of acceptable quality?”), item 7 (“Were the data on outcome collected using standardized methods of acceptable quality?”), and item 12 (“Positive, if the number of cases in the final multivariable was at least ten times the number of independent variables in the analysis.”), because of the different interpretation of the definitions of “standardized methods”, “acceptable quality”, and by miscalculating/interpretation of the number of cases in the final multivariable, respectively. Other disagreements were mostly due to differences in interpretation. All disagreements were resolved in a consensus meeting. Nine of the 13 prospective cohort studies [10,17,22,24,38,42,44–46] were considered to be high quality (> 6 items positive), as were the 2 retrospective cohort studies [9,47] (S3 Table).

### Risk Factors for Running Injuries; Overall and Injury Specific

The heterogeneity in study populations, in operationalization of both outcomes and risk factors, and time to follow-up prevented us from following a formal meta-analytical approach. Study populations varied from novice runners to recreational runner and competitive runners, outcomes from running-related injuries, overall injuries to lower leg overuse injuries and more localized injuries, e.g. Achilles Tendinopathy, back injuries (S4–S6 Tables). Follow-up time points varied from 8 weeks to 1 year (S2 Table). Across the studies different categories of independent variables were used with different cut-off points (S4–S6 Tables) or injured versus injured runners were compared using continuous values of risk factors (e.g. the mean age of injured runners was higher than the mean age of non-injured runners [46]). For these reasons we refrained from doing a meta-analysis. We therefore choose to present the results using a best evidence synthesis. Risk factors were divided into three categories: personal factors, running/training related factors and health and lifestyle factors (see S4–S6 Tables).

### Personal Factors; S4 Table

**Sex**. One low quality study [40] and five high quality studies [10,22,38,46,47] assessed sex as risk factor for running injuries. One high-quality studies [22] found men to have a significantly higher risk of running-related injuries than women, and particularly younger men (< 40 years) [47]. Thus there was limited evidence that men are at higher risk of running-related injuries.

**Age**. Four low-quality studies [39–41,43] and four high-quality studies [9,17,44,46] investigated the relationship between age and running injuries. Only one study found age to have a significant effect on running injuries: Wen et al. [17] showed that lower age was significantly protective against overall (not specified) overuse injury. Thus there was only limited evidence that lower age affects the risk of running-related injuries. Wen et al. [9] and Hirschmüller et al. [46] found higher age to be a significant risk factor for hamstrings injuries and midportion Achilles tendinopathy, respectively. This indicates that there is limited evidence that age affects the risk of hamstrings injuries and midportion Achilles tendinopathy.

**BMI**. Three low-quality studies [39,41,43] and three high-quality studies [9,38,46] examined BMI as a risk factor for running injuries. BMI was not found to have significant effect on injury risk in runners overall, but Wen et al. [9] found a higher BMI to be a risk factor for back injuries in women and a lower BMI to be a risk factor for foot injuries in men. Thus there was limited evidence that BMI is a risk factor for back injuries in women and for foot injuries in men.

**Height**. Four low-quality studies [39–41,43] and three high-quality studies [9,17,46] investigated height as a risk factor for running injuries. Wen et al. [9] found lower height in men to be a significant risk factor for foot injuries, indicating limited evidence.

**Weight**. Three low quality studies [39–41,43] and three high-quality [9,17,46] study investigated weight as a risk factor for running injuries. Wen et al. [9] found higher weight in women and lower weight men to be a risk factor for back injuries and foot injuries, respectively. In the same research group, Wen et al. [17] found higher weight to be protective against foot injuries. Thus there was limited evidence that higher weight in women and lower weight in men were risk factors for back and foot injuries, respectively. Furthermore, there was limited evidence that a heavier weight protects against foot injuries.

**Navicular drop**. One high-quality study [38] investigated the influence of navicular drop on running injuries. Bennett et al. [38] found runners with a high navicular drop (>10 mm) in the left or right foot were at greater risk for medial exercise-related leg pain. Also, a navicular drop of more than 10 mm in only the left foot was significantly associated with a higher risk of medial exercise-related leg pain. Thus there was limited evidence that navicular drop (> 10 mm) is a risk factor for running injuries.

**Intratendinous blood flow**. Only one high-quality study [46] investigated the influence of blood flow in the Achilles tendon on Achilles tendinopathy in runners. Runners with intratendinous microvessels (indicating primary neovascularization) were at greater risk of mid-portion Achilles tendinopathy. Thus there was limited evidence that impaired intratendinous blood flow is a risk factor for running injuries.

**Force distribution pattern**. Three low-quality studies [40,41,43] investigated force distribution patterns in relation to running injuries. Hesar et al. [40] found significantly less laterally directed force distribution at first metatarsal contact and forefoot flat, and significantly more medial directed force displacement in the forefoot contact phase, foot flat phase, and heel-off phase in runners without lower leg overuse injuries. These individuals also had a significantly quicker change in the center of force (COF) at forefoot flat, a lower force and loading underneath the lateral border of the foot, and a significantly lower directed force displacement of the COF at forefoot flat than did runners with lower leg injuries. Van Ginkel et al. [41] found a significant decrease in the total posterior–anterior displacement of the COF and a laterally directed force distribution underneath the forefoot at ‘forefoot flat’ as intrinsic gait-related risk factors for Achilles tendinopathy in novice runners. Thijs et al. [43] demonstrated that runners with a significantly higher vertical peak force underneath the second metatarsal and shorter time to the vertical peak force underneath the lateral heel were at higher risk for patellofemoral pain syndrome. In conclusion, there was limited evidence that a number of force distribution factors/patterns are risk factors for, or protective against, lower leg injuries, Achilles tendinopathy, and patellofemoral pain in runners [40,41,43].

**Alignment**. Three high-quality studies [9,17,44] investigated the influence of alignment on the occurrence of running injuries. In their prospective study, Wen et al. [17] found that runners in the group with the highest combined arch index were protective against, and runners in the group with the lowest leg difference were at higher risk for running injuries, respectively. In the retrospective study by the same research group [9], runners in the groups with the lowest left tubercle-sulcus angle and lowest combined (mean left and right) tubercle-sulcus angle were found to be at higher risk for ankle injuries. Runners in the groups with the lowest heel valgus, the highest heel valgus, and highest right arch index were found to be protective against knee injuries [17]. In this same prospective study, runners in the group with the highest left tubercle-sulcus angle and highest knee valgus were found to be significant at risk for shin injuries [17]. In subgroup analyses of this study, the highest heel valgus group was significant protective against foot injuries (expressed as injury incidence per 1000 miles running, or as injury incidence per 1000 hours running) [17]. In conclusion, there was limited evidence that a small difference in leg length is a risk factor for overall running injuries. There was also limited evidence that a large left tubercle-sulcus angle and a large knee varus are risk factors for shin injuries. Furthermore there was limited evidence that a low left tubercle-sulcus angle and combined (average of left and right) tubercle-sulcus angle are risk factors for ankle injuries and that several alignment factors are protective against running injuries.

### Running & Training Related Factors for Running Injuries; S5 Table

**Running experience**. Five high-quality studies investigated the relationship between running experience and running injuries [9,17,42,46,47]. Limited evidence was found that more running experience was a risk factor for overall running injuries [17]. There was also limited evidence that running with less (< 1 year) experience was protective for running injuries [47]. Limited evidence was found that more running experience was a risk factor for knee [42] and foot injuries [17].

**Training**. Five high-quality studies investigated the relationship between training factors and running injuries [9,17,42,46,47]. The prospective study of Wen et al. [17] found increased hours of running per week to be protective against overall injuries (expressed in terms of incidence per mileage or hours run). There was limited evidence that age < 40 years combined with running ≥ 6 times a week was a significant risk factor for running injury [47], as there was for age ≥ 40 years combined with running ≥ 6 times a week [47]. There was also limited evidence that age ≤ 40 years combined with running 1–3 times a week and running < 10 miles per week were significant protective factors for running injury [47], and an age ≥ 40 years combined with running 1–3 times a week was protective [47].

Van Middelkoop et al. [42] found that interval training was protective against knee injury in men. In contrast, the two high quality studies by Wen et al. [9,17] found more interval training to be a risk factor for shin injuries. The evidence for interval training being a risk or protective factor was limited. There was also limited evidence that increasing hours of running per week is protective against knee and foot injuries [17] and that a slower training pace was a risk factor for heel injuries [9].

**Surface**. Only one high-quality study [9] investigated the relationship between surface and running injuries. There was limited evidence that running time on concrete surface is protective against back and thigh injuries [9].

**Distance**. Four high-quality studies [9,42,44,46] analyzed running distance as independent variable for running injuries. There was limited evidence that higher weekly mileage is associated with hip and hamstrings injuries [9] and that a training distance of 0–40 km a week is protective against the incidence of calf injuries [42].

**Race participation**. One high-quality study [42] (= limited evidence) found the risk of running injuries to be higher in men who had participated in more than six races in the last year.

**Shoe use**. Two high-quality studies [9,17] analyzed the relationship between shoe use and running injuries. There was limited evidence that changing shoes more frequently was a risk factor for overall injuries [9] and limited evidence for using one pair of running shoes or alternating between two pairs versus alternating between more than two pairs of shoes as a risk factor for knee injuries [9]. Furthermore, limited evidence was found for a higher number of shoes as a risk factor for shin injuries [17].

### Health & Life-Factors Related for Running Injuries; S6 Table

**History of previous injury**. Four high-quality studies [17,38,42,46] investigated the relationship between running injuries and previous injuries. Bennett et al. [38] found that runners with a history of exercise-related leg pain for a month or a year were at greater risk of a relapse of exercise-related leg pain. Wen et al. [17] also found previous injuries to be a risk factor for running injuries. In the high-quality study of Van Middelkoop et al. [42], lower extremity injury in the previous 12 months was found to be a risk factor for running injury in men. In conclusion, there was strong evidence that previous injury is a risk factor for running injuries.

Van Middelkoop et al. [42] found that a lower extremity injury in the previous 12 months was a risk factor for a knee injury, and that an injury at another location (hip, groin, thigh, knee, ankle, or/and foot) was a risk factor for calf injury. None of the other studies identified risk factors for knee and/or calf injury. Bennett et al. [38] found that runners with a history of medial exercise-related leg pain lasting longer than 1 month were at greater risk of medial exercise-related leg pain. A history of old shin injuries was found to be a risk factor for shin injuries in one high-quality study [17]. A previous disorder of the Achilles tendon was a significant risk factor for midportion Achilles tendinopathy in one high-quality study [46]. In conclusion, there was limited evidence that previous injury is a risk factor for specific running injuries, namely, medial exercise-related leg pain, midportion Achilles tendinopathy, shin injuries, knee and calf injuries.

**Orthotic/inserts**. Two high-quality studies [9,47] investigated orthotic/inserts as a risk factor for running injuries. Both found wearing orthotics or using shoe inserts to be a risk factor for running injuries (moderate evidence). Wen et al. [9] found the use of shoe insert to be a risk factor for foot injuries, indicating limited evidence for this association.

### Sex Ratio

Five high-quality studies [10,22,24,45,47] analyzed data for men and women separately (see S7 Table). One study showed women to be at significantly lower risk of injuries overall than men [22]. Two studies showed men with a history of injury were at higher risk of running injuries than women with a similar history [22,45]. One high-quality study found the risk of injury to be higher in women than men if the women were older [10], had previously engaged in other sports activities [10], had the previous year participated in a marathon [45], had a weekly distance running of 48–63.8 km for the preceding 3 months [45], ran on concrete surface [45], and had running shoes that were 4- to 6-months old [24], with sex ratios of 1.4, 1.9, 2.0, 2.2, 4.2, and 4.9, respectively. Men were, in comparison with women, at greater risk of injury if they restarted running [10], had less than 2 years’ running experience [45], had a weekly running distance of 32–47.8 km [45] or had a weekly running distance > 64 km [45], with a sex ratio of 0.7, 0.7, 0.7 and 0.4, respectively.

## Discussion

The purpose of this study was to synthesize current evidence on determinants of running-induced injuries of the leg in adults and to determine sex differences in risk profile for running injuries. We found strong and moderate evidence that previous leg injury and use of orthotics/inserts increase the risk of leg injuries, respectively. Furthermore, there was only limited (one high-quality study) or no (one/two low-quality studies) evidence for other potential risk factors for running injuries (overall and injury specific).

Analysis of the sex ratios showed that women are at lower risk of running injuries than men. Factors that increased the risk of running-related injuries in women were older age, previous participation in non-axial sports (e.g. cycling, swimming, etc.), participating last year in a marathon, running on concrete, a longer weekly running distance (48–63.8 km) and wearing running shoes for 4 to 6 months. Men were at greater risk of such injuries if they restarted running, had a history of previous injuries, a running experience of 0–2 years, had a weekly running distance between 32–47.8 km, and having a weekly running distance more than 64 km per week.

Running injuries have a multifactorial origin that can be subdivided into personal, running/training, and health and/or lifestyle factors [5,14,15]. These factors can reinforce each other and their influence may also be mediated by cultural or societal factors [49]. The importance of each factor, and hence its contribution to the risk of symptoms and injuries, varies among individuals and running environments. Personal factors investigated in this review focused on sex, age, anthropometric, and biomechanical factors; psychosocial factors were not investigated as risk factors for running injuries. Psychosocial factors seem to have a role in musculoskeletal disorders [49–51] and thus future studies should investigate their role in running-related leg injuries.

Most running injuries are due to overuse [7], but only Wen et al. [9,17] and Bennett et al. [38] included or excluded overuse/acute injuries in their definition of injury, respectively. Overuse injuries of the musculoskeletal system generally occur when a structure is repeatedly exposed to loading forces. Forces lower than the threshold associated with acute injury ultimately lead to fatigue of that specific structure [52,53]. There is no standard definition of overuse running injury [8,54], but it should minimally include a musculoskeletal ailment that can be attributed to running and that causes a restriction of running speed, distance, duration, or frequency for at least a week [8]. Of the articles included in our review, that of Buist et al. [10,22] used definitions “any musculoskeletal pain of the lower limb or back causing a restriction in running for at least one day [10] or one week [22]” that matches the most with these criteria. The other studies did not define the period during which injury restricted running. Future research should use the definition of running injuries used by Buist et al. [10,22] or include a minimal time frame of running restriction when defining running-related injuries.

To our knowledge, this is the third review that systematically examined risk factors for running injuries. Five reviews of running injuries have been published in the past [4,5,14,15,19], and three of these narrative studies were published more than 20 years ago [5,14,15]. The most recent systematic reviews were published in 2007 [4] and 2014 [19]. Van Gent et al. [4] found strong evidence that a long training distance per week in men and previous injuries were risk factors for injuries; however, a long training distance per week was a protective factor for knee injuries. Although we also found previous injury to be a risk factor for running-related injuries, the variety in the other results can be explained by differences in the studies included. Seventeen articles, dating from 1982 to 2006, were included [4]: 10 studies were published after 2006 and were therefore not included in the study of Van Gent et al. [4]. As we used a minimal follow-up time of 1 month and an age of >18 years as inclusion criteria, the studies of Walter et al. [18] and Satterthwaite et al. [16] were not included in our review. The finding of Van Gent et al. [4] that longer training distance per week is a protective against knee injuries could not be confirmed because studies providing evidence for this association were not included in our review.

The recent published review by Saragiotto et al. [19] included only prospective studies which mentioned running or runners in the abstract/title. Moreover, articles that studied risk factors for specific injuries (e.g. medial tibial stress syndrome) were excluded in their systematic review. Furthermore, Saragiotto et al. [19] included all categories of runners, this in contrast with our study population consisting of novice runners, long-distance runners, both recreational and/or competitive. In their study [19] also pooling of data was not possible due to the large heterogeneity of the statistical methods used across studies. However, although they did not perform a best evidence synthesis and used different inclusion and exclusion criteria, the conclusion that previous injury is a risk factor for running injuries was the same as in our study.

### Risk Factors for Running Injury

We decided to classify the different risk factors for running injuries according to the existing literature of systematic reviews (personal, running/training, health and lifestyle) [4,14,15], to facilitate comparison between the reviews. However, applying a public health approach to sports injury prevention as described by Finch [55], conceptualizing risk factors as modifiable and nonmodifiable provides additional insight [56]. Modifiable risk factors associated with running injuries provide the base for developing running injury prevention interventions, whereas nonmodifiable risk factors are important for risk stratification and targeted prevention [56].

### Nonmodifiable Risk Factors for Running Injuries

**History of injury**. Previous injury was consistently associated with running injuries and especially in men. The lack of association between previous injury and running injuries in women might be because most of the included studies investigated female novice runners with minimal running experience and few injuries in the past [10,22,24,39–41,43].

It is not clear whether a high rate of re-injury is due to incomplete healing of the original injury, an uncorrected biomechanical problem, or recall bias and/or the definition of the injury. Previous lower extremity injuries that have healed completely (i.e., the return of full, pre-injury joint range of motion, musculoskeletal strength, and proprioception) should not increase the risk of a subsequent lower extremity injury [57]. However, injuries that give rise to permanent structural or biomechanical malfunction and/or dysfunctional coordination increase the risk of future running injuries [58]. In our review, three high-quality studies [22,42,45] found a history of previous leg injury to be a risk factor in men. However, the definition of “previous injury” differed in the various studies, in terms of its nature (e.g. acute or gradual onset), whether it is running related or not, when it occurred and how long it lasted. It is essential to know the extent and characteristics of recovery from a previous injury [57]. Lastly, in most studies participants were asked about injuries in the previous year, which means that recall bias could be a problem.

In conclusion, previous (running) leg injury seems an important risk factor for running injuries. Further research should focus on a clear definition of “previous (running) injury” and should more focus on recovery processes to judge the possibility of re-injury including the time of occurrence, and on minimizing recall bias by reducing the time frame of recall.

### Modifiable Risk Factors for Running Injuries

**Training**. Overuse running injuries are suggested to be the result of training errors [8] and our results confirm this. On the basis of this review, it seems that the ideal training intensity has not yet been established. Runners with a high training frequency and/or running distance appeared to be more susceptible to overuse injuries, especially those runners who have no running experience and, seemingly contradictory, runners who are experienced and who have run, perhaps long distances, for a longer time. Van Gent et al. [4] found strong evidence that men with a higher weekly training frequency were more prone to running injuries. However, running only once a week could lead to overuse injuries, especially in women [24]. This is probably because running stresses the musculoskeletal system [8], which does not have time to adapt to this type of exercise because of the low frequency of running.

In conclusion, overuse running injuries should be prevented by optimizing and personalizing training, bearing in mind the (limited) evidence that running/training-related factors influence the risk of injury.

**Orthotic/insert**. Foot orthoses are widely used to treat existing pathological conditions and to prevent overuse injuries [59]. They function in two ways: 1) the insert acts as a cushion that absorbs shock transmitted to the lower limb, and 2) they compensate for biomechanical deficiencies of the foot, such as excessive pronation and differences in leg length [60]. Most findings of this review contradict these statements. McKean et al. [47] and Wen et al. [9] showed that runners with orthotic/inserts were at higher risk of running injuries, although it is possible that runners who are more prone to injury are given orthotic/inserts earlier. However, given the findings about the role of the navicular drop [22], alignment [9,17], and force distribution [40,41] in running-related injuries, it is doubtful that compensating biomechanical deficiencies with an orthotic/insert is effective in preventing running injuries. In conclusion, orthotics/inserts do not seem useful to compensate for biomechanical deficiencies.

### Sex Differences

Differences between the health of men and women are a major concern to European health authorities [20]. Only five high-quality studies [10,22,24,45,47] investigated the effect of runner’s sex on the risk of running injuries. However, given the small number of studies that investigated this, it was not possible to establish sex-specific profiles for risk factors.

Two high-quality studies investigated the relation between previous injury and running injuries and presented data for men and women separately, so that it was possible to calculate a sex ratio. When the criteria of Van Tulder [33] were used to determine the level of evidence for sex differences, two studies [22,47] provided moderate evidence that men (< 40 year) had a higher risk of running-related injuries and two studies [22,45] provided moderate evidence that men had a higher risk of running-related injuries when having a previous injury; the other studies did not provide evidence of sex-related differences in risk of running injuries. However, physical therapists, sports physicians, etc. can provide sex-specific advice for the prevention of running injuries, and trainers and coaches can tailor their training advice to individual runners. More prospective longitudinal studies are necessary and should analyze data for men and women separately, in order to obtain evidence-based, sex-specific risk profiles [20,61].

### Risk of Bias & Study Limitations

As risk factors were operationalized as dichotomous, ordinal, or even continuous variables, it was not possible to calculate a meaningful pooled summary of outcomes. Moreover, conclusions made after data pooling might have been of limited value given the heterogeneity in definition of running injury in the various studies.

Quality scoring systems are used in an attempt to address possible methodological shortcomings that could threaten the validity of study results [30]. We created our quality scale based on the lists used by the Cochrane Collaboration to assess cohort studies [27] and on lists used in previous studies [28–30]. One of these lists [29] was quantified by West et al. [62] in a study that evaluated quality-rating systems for observational studies. The scoring list of Ariëns et al. [29] scored positive on six and partially positive on one out of nine domains for assessing study quality [62]. While the usefulness of quality control is disputed [62] as it is difficult to determine how to weight each item in an overall quality score, sum scores are considered helpful in a systematic review for distinguishing between studies with a low or a high risk of bias [62,63]. We evaluated the quality of the included studies in order to gain insight into the risk of bias and therefore to enable us to draw meaningful conclusions. A point of concern is that many of the included studies did not clearly describe the participation rate of the target group, which limits the generalizability of findings [64].

This study had some limitations. All included studies, prospective and retrospective, were assessed using the same quality list. Because it would be better to adjust the list for a retrospective design, a second quality analysis was done for the two retrospective studies reviewed [9,47], such that item 2 (“participation rate is at least 80% from the identified target group”) and 3 (“the participation rate at main moment of follow-up is at least 80% or the nonresponse is not selective”) were scored as “not applicable” in the scoring list. This did not influence the quality score of these articles (both remained high quality), and therefore had no influence on the results of our best evidence syntheses.

By our inclusion criteria (e.g. long-distance runners recreational and/or competitive) for selecting the original studies, a broad spectrum in the type of runners (novice, track and field, etc.) was selected. When the inclusion criteria were more strictly defined, our results could be presented stratified for each group of runners. However, the number of studies per type of runners would be too small to give useful information and by choosing a broader spectrum of type of runners, our results are more generalizable to the total adult running population.

Although we performed an extensive literature search, it is likely that both selection and publication bias influenced the results. Future research, in which running injury is uniformly defined, may indicate whether the factors found in our review are true risk factors.

### Conclusion and Implications

More high-quality studies of risk factors for running injuries are needed before strong conclusions can be drawn about the relevance of specific risk factors. Furthermore, consensus must be achieved about the definition of running injuries, and large cohort studies are needed to investigate different types (biomechanical, hormonal, psychological, etc.) of risk factors with emphasis on potential differences between men and women. To minimize bias, future studies should pay attention to recall of previous running injuries, follow-up time, and the participation rate.

This review found strong evidence that previous leg injury is a risk factor for running-related leg injuries. Some sex-specific risk factors were identified, but not enough studies investigated differences between men and women to obtain more definite results.

Running injuries seem to have a multifactorial origin, but on the basis of our findings, efforts to prevent injury should focus on runners, especially men, with a history of running injuries and provide customized training and/or specific exercises. The use of orthotics/inserts should be discouraged.

## Supporting Information

S1 PRISMA Checklist (DOCX)

S1 Fig Flow Chart of the search of articles. (TIFF)

S2 Fig PRISMA 2009 flow diagram. (DOCX)

S1 Table Quality assessment check list [27–30]. (DOCX)

S2 Table Study Characteristics. (DOCX)

S3 Table Results of the risk of bias assessment. (DOCX)

S4 Table Significant personal risk- & protective factors for running injuries. (DOCX)

S5 Table Significant running/training-related risk factors for running injuries. (DOCX)

S6 Table Significant health & lifestyle factors related for running injuries. (DOCX)

S7 Table Risk factors for running injuries with sex ratio. (DOCX)

S1 Appendix Search terms PubMed. (DOCX)
