# Supplementary material for: Injuries in Runners; A Systematic Review on Risk Factors and Sex Differences
Source: PLoS One. 2015 Feb 23;10(2):e0114937. doi: 10.1371/journal.pone.0114937 (PMC4338213; doi:10.1371/journal.pone.0114937)
Supplement: S2 Fig — (DOCX) [file pone.0114937.s003.docx]

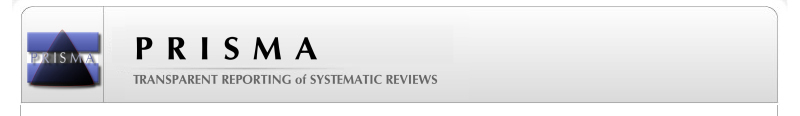
**PRISMA 2009 Flow Diagram**

Studies included in quantitative synthesis (meta-analysis)
(n = 0 )

Studies included in qualitative synthesis
(n = 15 )

Full-text articles excluded, with reasons
(n =2 )

Full-text articles assessed for eligibility
(n = 17 )

Records excluded
(n = 383 )

Records screened
(n = 400 )

Records after duplicates removed
(n = 400 )

Additional records identified through other sources
(n = 0 )

## Identification

## Eligibility

## Included

## Screening

Records identified through database searching
(n = 633 )
