# Supplementary material for: Injuries in Runners; A Systematic Review on Risk Factors and Sex Differences
Source: PLoS One. 2015 Feb 23;10(2):e0114937. doi: 10.1371/journal.pone.0114937 (PMC4338213; doi:10.1371/journal.pone.0114937)
Supplement: S1 Table — (DOCX) [file pone.0114937.s004.docx]

**Table S1. Quality assessment check list [27-30]**

**Study objective**

1. Positive, if the main features of the study population were described (sampling frame and distribution of the population according to age and sex).
2. Positive, if the participation rate was at least 80% of the identified target group.
3. Positive, if the participation rate at the main moment of follow-up was at least 80% or if the nonresponse is not selective (data shown).

**Exposure measurements**

1. Positive, if the study population consisted of subjects without symptoms or if data on symptoms are included in the statistical analysis.
2. Positive, if data on system factors, running/training-related factors, and/or health and lifestyle factors were collected using standardized methods of acceptable quality.

**Outcome assessments**

1. Positive, if the follow-up period was at least 1 year.
2. Positive, if outcome data were collected using standardized methods of acceptable quality.

**Analysis and data-presentation**

1. Positive, if the measures of association were presented (OR/RR), including confidence intervals and numbers in the analysis.
2. Positive, if the analysis was controlled for confounding or effect modification: system factors.
3. Positive, if the analysis was controlled for confounding or effect modification: running/training related factors.
4. Positive, if the analysis was controlled for confounding or effect modification: health and life-factors.
5. Positive, if the number of cases in the final multivariable was at least ten times the number of independent variables in the analysis.
