# Supplementary material for: Injuries in Runners; A Systematic Review on Risk Factors and Sex Differences
Source: PLoS One. 2015 Feb 23;10(2):e0114937. doi: 10.1371/journal.pone.0114937 (PMC4338213; doi:10.1371/journal.pone.0114937)
Supplement: S2 Table — (DOCX) [file pone.0114937.s005.docx]

**Table S2. Study Characteristics**

| **Author, year of publication** | **Study design, size and follow up period** | **Population characteristics** | | | | | | | | **Injury definition** | **Incidence (%)** |
| --- | --- | --- | --- | --- | --- | --- | --- | --- | --- | --- | --- |
|  |  | **Age (yr)**  **(mean ± sd)** | | **Sex (%)** | | **BMI**  **(kg/m2)** | **No included/ analyzed (%)** | **Running type** | |  |  |
| Bennett et al., 2012 [38] | Prospective cohort, n=77  Cross-country season | N.F. | M: 52.5  W: 47.5 | | 13.6% <18.5 | | 77/59 (77%) | | Cross-country athletes | Exercise-related leg pain: pain located in the anterior, medial, posterior, or lateral leg not associated with a traumatic injury. | Overall: 44.1  M; 41.9  W: 46.4 |
| Hirschmüller et al., 2012 [46] | Prospective cohort, n=634  1 year | 43.2 ± 11.0 (range 19 - 74) | M: 66.7  W: 33.3 | | 23.0 ± 2.0 (range 16.0 – 35.8) | | 634/427 (67%) | | Runners from 23 national running events | Midportion Achilles tendinopathy: pain localized 2-6 cm proximal to the insertion and at least two of the following minor criteria reported: palpable thickening of the tendon, tenderness of the Achilles tendon, or Achilles tendon pain at the beginning of physical activity. | Injury specific: 14.3 |
| Thijs et al., 2011 [39] | Prospective cohort, n=77  10 weeks | 38 ± 9 | W: 100 | | 24.6 ± 2.9 | | 77/77 (100%) | | Female novice recreational runners | Patellofemoral dysfunction syndrome: Characteristic history and symptoms involved retropatellar pain: typically dull and diffuse around the patellofemoral joint, during and/or after activities such as running, squatting, kneeling, going up and down stairs, cycling, prolonged sitting with the knee in flexion, or rising from a seated position. Other symptoms involved stiffness and swelling, giving way, and crepitus at the knee. On clinical assessment, the participants had to exhibit two of the following clinical criteria: (1) pain while compressing the patella against the femoral condyles with the knee in full extension (2) tenderness of the medial and/or lateral posterior surface of the patella on palpation (3) a painful resisted knee extension and/or (4) pain at the patellofemoral joint when isometrically contracting the quadriceps muscle against suprapatellar resistance with the knee in 15o of flexion. | Injury specific: 20.8 |
| Buist et al., 2010 [10] | Prospective cohort, n=875  8 weeks | 43.7 ± 9.5  M: 46.5 ± 9.4  F: 42.3 ± 9.2 | M: 32.9  W: 67.1 | | 24.9 ± 3.3  M: 25.9 ± 3.2  F: 24.4 ± 3.2 | | 875/629 (72%) | | Novice and recreational runners | Running-related injury: any musculoskeletal pain of the lower limb or back causing a restriction in running (mileage, pace or duration) for at least 1 day. | Overall: 25.9  M: 31.4  W: 23.2 |
| Buist et al., 2010 [22] | Prospective cohort, n=532  8 or 13 weeks | M: 42.3 ± 9.9  F: 37.9 ± 9.9 | M: 42.5  W: 57.5 | | M: 25.9 ± 3.3  F: 24.2 ± 3.4 | | 532/532 (100%) | | Novice runners | Running-related injury: running-related musculoskeletal pain of the lower extremity or back causing a restriction of running for at least 1 week, that is, 3 scheduled consecutive training sessions. | Overall: 20.6 |
| Hesar et al., 2009 [40] | Prospective cohort, n=131  10 weeks | 39.09 ± 10.3 | M: 15.3  W: 84.7 | | Weight (kg): 70.33 ± 11.31  Height (cm):  168.47 ± 7.77 | | 131/131 (100%) | | Novice runners | Lower leg overuse injuries | Overall: 20.6  M: 25.0  W: 19.8 |
| Van Ginckel et al., 2009 [41] | Prospective cohort, n= 129  10 weeks | 39 ± 10 | M: 14.7  W: 85.3 | | 24.8 ± 3.5 | | 129/63 (49%) | | Novice runners | Achilles Tendinopathy: an insidious, gradual onset of mid-portion pain, palpated tenderness along the tendon, (morning) stiffness, tenderness and pain on exertion. | Overall: 53.5  Injury specific: 7.8 |
| Van Middelkoop et al., 2008 [42] | Prospective cohort, n= 1500  1 month | 43.8 ± 9.6 | M: 100 | | 23.5 ± 2.1  BMI > 25:  15.1% | | 1500/694 (46%) | | Recreational/ amateur runners | Running injury: a self-reported ‘’injury on muscles, joints, tendons and/or bones of the lower extremities (hip, groin, thigh, knee, lower leg, ankle foot and toe) that the participant attributed to running’’. The problem had to be severe enough to cause a reduction in the distance, speed, duration or frequency of running. | Overall: 28.1 |
| Thijs et al., 2008 [43] | Prospective cohort, n= 129  10 weeks | 37 ± 9.5 | M: 12.7  W: 87.3 | | 25 ± 3 | | 129/102 (79%) | | Novice recreational runners | Patellofemoral pain (PFP): a characteristic history and symptoms of PFP syndrome and exhibit two of the following criteria on assessment: pain on direct compression of the patella against the femoral condyles with the knee in full extension; tenderness of the posterior surface of the lateral or medial rim of the patella on palpation; pain on resisted knee extension; or pain with isometric quadriceps muscle contraction against suprapatellar resistance with the knee in 15° of flexion. In addition, subjects had to have negative findings in the examination of knee ligaments, menisci, bursae, synovial plicae, Hoffa’s fat pad, iliotibial band, the hamstrings, quadriceps and patellar tendons and their insertions. | Injury specific: 16.7 |
| McKean et al., 2006 [47] | Retrospective survey, n=2886  1 year | > 40 years: 34% | N.F. | | N.F. | | 2886/2886 (100%) | | Runners participating in Hood to Coast Relay | Running injury: an event that affected the athlete’s ability to train or race over the previous year. | Overall: 46.3 |
| Lun et al., 2004 [44] | Prospective cohort, n=153  6 months | 38.0 | M: 50.6  W: 49.4 | | N.F. | | 153/87 (57%) | | Recreational runners | Lower limb injury: any musculoskeletal symptom of the lower limb that required a reduction or stoppage of normal training. | Overall: 79.3  M: 79.5  W: 79.1 |
| Taunton et al., 2003 [24] | Prospective cohort, n= 844  13 weeks | M: 12.3% <30, 51.5% 31 – 49, 19.1% 50 – 55, 17.2% >56.  F: 18.6% <30, 63.6% 31 – 49, 11.5% 50 – 55, 6.3% >56. | M: 24.4  W: 75.6 | | M: 1.0% <19, 55.1% 20 – 26, 41.0% >26.  F: 4.3% <19, 69.8% 20 – 26, 16.7% > 26. | | 844/840 (100%) | | Recreational runners interested in either completing the 10 km race distance (novice runners) or improving their race time (intermediate runners). | Injury: 1, pain only after exercise; 2, pain during exercise, but not restricting distance or speed; 3, pain during exercise and restricting distance and speed; 4, pain preventing all running. A runner was classified as being injured if they experienced at least a grade 1 injury (pain only after exercise). Diagnoses from participants that did not consult a qualified doctor or physiotherapist were not included. | Overall: 29.5 |
| Wen et al., 1998 [17] | Prospective cohort, n= 355  32 weeks | 41.8 ± 10.8 | M: 42.0  W: 58.0 | | M: Weight (kg): 79.3 ± 11.7  Height (cm):  176.8 ± 6.3  F: Weight (kg): 64.1 ± 12.3  Height (cm):  164.3 ± 7.3 | | 355/255 (72%) | | Runners participating in a training program for a marathon | Running injury: answering yes to having had ‘injury or pain’ to an anatomical part; answering yes to having had to stop training, slow pace, stop intervals, or otherwise having had to modify training; and a ‘gradual’ versus ‘immediate’ onset of the injury or a self-reported diagnosis that is generally considered an overuse injury. | Overall: 35.3 |
| Wen et al., 1997 [9] | Retrospective cohort, n= 355  12 months | 41.1 ± 10.6 (range: 21 -78) | M: 43.8  W: 56.2 | | M: Weight (kg): 81.0 ± 13.4 (range: 59 – 128)  Height (cm): 177.3 ± 6.8 (range: 163 – 193)  F: Weight (kg): 64.0 ± 13.4 (range: 43 – 146)  Height (cm): 164.3 ± 7.3 (range: 150 - 183) | | 355/304 (86%) | | Experienced runners enrolling in a marathon training program | Running injury: subject answered “yes” to having had “injury or pain” to that anatomic part; and answered “yes” to having had to stop training, or to have to slow pace, stop intervals, or otherwise to have had to modify training; and the onset of the injury was “gradual” (versus “immediate”), or his or her diagnosis (self-reported) was one that is generally considered an overuse injury. | Overall: 44.7 |
| Macera et al., 1989 [45] | Prospective cohort, n= 966  12 months | M: 41.6 ± 9.5 (range: 13 – 75)  F: 36.1 ± 8.2 (range: 22 – 64) | M: 83.2  W: 16.8 | | M: 23.0 ± 2.2 (range: 16.5 – 31.0)  F: 25.8 ± 2.4 (range: 20.2 – 34.6) | | 966/583 (60%) | | Habitual runners | Running-related lower-extremity injury: A self-reported ‘’muscle, joint or bone problem/injury’’ of the lower extremities (foot, ankle, Achilles tendon, calf, shin, knee, thigh, or hip) that the participant attributed to running. The problem had to be severe enough to cause a reduction in weekly distance, a visit to a health professional, or the use of medication. | Overall: 51.5  M: 52.0  W: 49.0 |

NF, not found: BMI, body mass index: W, women: M, men
