# Supplementary material for: Injuries in Runners; A Systematic Review on Risk Factors and Sex Differences
Source: PLoS One. 2015 Feb 23;10(2):e0114937. doi: 10.1371/journal.pone.0114937 (PMC4338213; doi:10.1371/journal.pone.0114937)
Supplement: S3 Table — (DOCX) [file pone.0114937.s006.docx]

**Table S3. Results of the risk of bias assessment**

|  |  | **1** | **2** | **3** | **4** | **5** | **6** | **7** | **8** | **9** | **10** | **11** | **12** | **Total score (n)** | **Total score (%)** |
| --- | --- | --- | --- | --- | --- | --- | --- | --- | --- | --- | --- | --- | --- | --- | --- |
| 1 | Bennett et al., 2012 [38] | + | - | + | + | + | - | - | + | + | - | + | - | 7 | 58 |
| 2 | Hirschmüller et al., 2012 [46] | + | ? | - | + | + | + | - | + | + | - | + | + | 8 | 67 |
| 3 | Thijs et al., 2011 [39] | + | ? | + | + | + | - | + | - | + | - | - | - | 6 | 50 |
| 4 | Buist et al., 2010 [10] | + | - | - | + | + | - | - | + | + | + | + | + | 8 | 67 |
| 5 | Buist et al., 2010 [22] | + | + | + | + | + | - | - | + | + | + | + | ? | 9 | 75 |
| 6 | Hesar et al., 2009 [40] | + | ? | + | + | + | - | ? | + | + | - | - | - | 6 | 50 |
| 7 | Van Ginckel et al., 2009 [41] | + | ? | - | + | + | - | ? | + | + | - | + | - | 5 | 42 |
| 8 | Van Middelkoop et al., 2008 [42] | + | - | + | + | + | - | - | + | + | + | + | + | 9 | 75 |
| 9 | Thijs et al., 2008 [43] | + | ? | - | + | + | - | + | - | + | - | - | - | 5 | 42 |
| 10 | McKean et al., 2006 [47] | + | - | + | + | + | - | + | + | + | + | + | + | 10 | 83 |
| 11 | Lun et al., 2004 [44] | + | ? | + | + | + | - | - | - | + | + | + | - | 7 | 58 |
| 12 | Taunton et al., 2003 [24] | + | + | + | - | + | - | + | + | + | + | + | + | 10 | 83 |
| 13 | Wen et al., 1998 [17] | + | - | + | + | + | - | - | + | + | + | + | + | 9 | 75 |
| 14 | Wen et al., 1997 [9] | + | - | + | + | + | + | - | - | + | + | - | - | 7 | 58 |
| 15 | Macera et al., 1989 [45] | + | - | ? | + | + | + | - | + | + | + | + | - | 8 | 67 |

+ Well-described and well-performed item; - item was described but not well performed; ? item was unclear due to insufficient available information.
