# Supplementary material for: Injuries in Runners; A Systematic Review on Risk Factors and Sex Differences
Source: PLoS One. 2015 Feb 23;10(2):e0114937. doi: 10.1371/journal.pone.0114937 (PMC4338213; doi:10.1371/journal.pone.0114937)
Supplement: S4 Table — (DOCX) [file pone.0114937.s007.docx]

**Table S4. Significant personal risk- & protective factors for running injuries**

| **Independent variable** | **MQ** | **Author** | **Injury** | **Specification of independent variable** | **Outcome (95% CI)** |
| --- | --- | --- | --- | --- | --- |
| Sex | HQ | Buist et al., 2010 [22] | Running-related injury (RRI) | M, 42.5% | HR= 1.5 (P = 0.04) |
|  | HQ | McKean et al., 2006 [47] | Running injury | M < 40 yrs. | OR= 1.28 (1.06 – 1.54)† |
| Age | HQ | Wen et al., 1998 [17] | Overall injuries | Lower age (group: miles)‡ | RR= 0.39 (0.15 – 0.97)† |
|  | HQ | Wen et al., 1997 [9] | Hamstrings injuries | Higher age | P= 0.019 |
|  | HQ | Hirschmüller et al., 2012 [46] | Midportion Achilles tendinopathy (MPT) | Higher age | P< 0.05 |
| BMI | HQ | Wen et al., 1997 [9] | Back injuries | Higher BMI (women) | P= 0.009 |
|  |  |  | Foot injuries | Lower BMI (men) | P= 0.045 |
| Height | HQ | Wen et al., 1997 [9] | Foot injuries | Shorter height (men) | P= 0.033 |
| Weight | HQ | Wen et al., 1997 [9] | Back injuries | Higher weight (women) | P= 0.002 |
|  |  |  | Foot injuries | Lower weight (men) | P= 0.011 |
|  | HQ | Wen et al., 1998 [17] | Foot injuries | Higher weight§ | RR= 0.94 (0.89 – 0.99)† |
| Navicular drop (ND) | HQ | Bennett et al., 2012 [38] | Medial exercise-related leg pain (ERLP) | Left ND >10 mm (hyperpronation)  Right or left ND >10 mm (hyperpronation) | OR= 5.3 (1.2- 22.9)  OR= 4.4 (1.0- 18.9) |
| Intratendinous blood flow | HQ | Hirschmüller et al., 2012 [46] | Midportion Achilles tendinopathy (MPT) | Neovascularization I^o^ | OR= 6.9 (2.6 – 18.8)† |
| Mediolateral force ratios | LQ | Hesar et al., 2009 [40] | Lower leg overuse injuries (LLOI) | First metatarsal contact; ratio 1  First metatarsal contact; ratio 2  First metatarsal contact; ratio 3  First metatarsal contact ratio 8  Forefoot flat; ratio 1  Forefoot flat; ratio 3  Initial contact phase ratio 8 | OR= 0.63 (0.42- 0.95)  OR= 0.64 (0.42- 0.96)  OR= 0.63 (0.42- 0.95)  OR= 0.58 (0.38- 0.89)  OR= 0.63 (0.41- 0.98)  OR= 0.62 (0.40- 0.96)  OR= 0.62 (0.39- 1.00) |
|  | LQ | Van Ginckel et al., 2009 [41] | Achilles Tendinopathy (AT) | Forefoot flat; ratio 2  Less force ratios at foot flat phase ratio 2 | P= 0.009  P = 0.045 |
| Mediolateral displacement of the centre of force | LQ | Hesar et al., 2009 [40] | Lower leg overuse injuries (LLOI) | x-comp heel off  x-comp forefoot contact phase  x-comp foot flat phase  x-comp forefoot push-off phase | OR= 0.76 (0.64- 0.92)  OR= 0.41 (0.23- 0.74)  OR= 0.58 (0.38- 0.90)  OR= 1.64 (1.07- 2.51) |
| Velocity mediolateral displacement of the centre of force | LQ | Hesar et al., 2009 [40] | Lower leg overuse injuries (LLOI) | Forefoot flat | OR= 0.50 (0.27- 0.92) |
| Anteroposterior displacement of the centre of force | LQ | Hesar et al., 2009 [40] | Lower leg overuse injuries (LLOI) | Forefoot flat | OR= 1.47 (1.12- 2.77) |
|  | LQ | Van Ginckel et al., 2009 [41] | Achilles Tendinopathy (AT) | Total displacement (mm)  Less displacement at last foot contact (mm)  Less displacement during forefoot push off phase (mm) | P= 0.007  P = 0.008  P = 0.033 |
| Absolute force-time integral underneath metatarsal heads | LQ | Hesar et al., 2009 [40] | Lower leg overuse injuries (LLOI) | Metatarsal head 5 | OR= 1.72 (1.13- 2.61) |
| Vertical peak force underneath foot | LQ | Hesar et al., 2009 [40] | Lower leg overuse injuries (LLOI) | Metatarsal head 5 | OR= 1.66 (1.08- 2.54) |
|  | LQ | Thijs et al., 2008 [43] | Patellofemoral pain (PFP) | Higher force underneath metatarsal head 2  Higher force underneath metatarsal head 3  Higher force underneath lateral heel | P= 0.016  P= 0.026  P= 0.034 |
| Time to peak force | LQ | Van Ginckel et al., 2009 [41] | Achilles Tendinopathy (AT) | At medial heel – less seconds | P = 0.032 |
| Time to vertical peak force relative to time of foot contact | LQ | Thijs et al., 2008 [43] | Patellofemoral pain (PFP) | Less time at medial heel  Less time at lateral heel | P= 0.016  P= 0.037 |
| Alignment measurements | HQ | Wen et al., 1998 [17] | Overall injuries | Low leg length difference (group: hours)‡  Higher combined arch index (group: hours)‡ | RR= 1.96 (1.07 – 3.58)†  RR= 0.0 (0.0 – 0.37)† |
|  |  |  | Knee injuries | Low heel valgus (group: miles)‡  High heel valgus (group: miles)‡  Higher right arch index (group: hours)‡ | RR= 0.08 (0.01 – 0.74)†  RR= 0.09 (0.01 – 0.81)†  RR= 0.11 (0.01 – 0.90)† |
|  |  |  | Shin injuries | High left tubercle-sulcus angle (group: miles)‡  Higher knee varus (group: miles)‡ | RR= 11.02 (2.00 – 60.86)†  RR= 1.09 (1.03 – 1.15)† |
|  |  |  | Foot injuries | Higher heel valgus (group: miles)‡  Higher heel valgus (group: hours)‡ | RR= 0.76 (0.58 – 0.98)†  RR= 0.74 (0.58 – 0.94)† |
|  | HQ | Wen et al., 1997 [9] | Ankle injuries | Lowest left tubercle-sulcus angle  Lowest combined tubercle-sulcus angle | P = 0.02  P= 0.049 |

† Represents adjusted OR, HR or RR
§ RRs were calculated dividing the number of injured runners by the total number of runner-weeks accumulated (relative incidence ratios) (n = 255)
‡ RRs were obtained from special subgroups in which information on distances run (miles) and time spent running (hours) was measured (total n = 108)
M, men: BMI, Body mass index: ND, Navicular drop: CI, Confidence interval: OR, Odds ratio: HR, Hazard ratio: RR, Relative risk: MQ, Methodological quality: HQ, High quality: LQ, Low quality
