# Supplementary material for: Injuries in Runners; A Systematic Review on Risk Factors and Sex Differences
Source: PLoS One. 2015 Feb 23;10(2):e0114937. doi: 10.1371/journal.pone.0114937 (PMC4338213; doi:10.1371/journal.pone.0114937)
Supplement: S5 Table — (DOCX) [file pone.0114937.s008.docx]

**Table S5. Significant running/training-related risk factors for running injuries**

| **Independent variable** | **MQ** | **Author** | **Injury** | **Specification of independent variable** | **Outcome (95% CI)** |
| --- | --- | --- | --- | --- | --- |
| Running experience | HQ | McKean et al., 2006 [47] | Running injury | < 40 yrs group: Running less than 1 year (↓ risk) | P= 0.01 |
|  | HQ | Wen et al., 1998 [17] | Overall injuries | Greater experience§ | RR= 1.88 (1.16 – 3.05)† |
|  |  |  | Foot injuries | Greater experience§ | RR= 1.09 (1.03 – 1.15)† |
|  | HQ | Van Middelkoop et al., 2008 [42] | Incident knee injury | Running experience; 15+ years | OR= 2.56 (1.22 – 5.34)† |
|  | HQ | McKean et al., 2006 [47] | Running injury | < 40 yrs group   - 6+ runs p/w - 1- 3 runs p/w (↓ risk) - Running < 10 miles per week (↓ risk)   ≥ 40 yrs group   - 6+ runs p/w - 1- 3 runs p/w (↓ risk) | P< 0.05  P< 0.05  P< 0.05  P= 0.01  P= 0.001 |
|  | HQ | Wen et al., 1998 [17] | Overall injuries | Increased hours/week (group: miles)‡  Increased hours/week (group: hours)‡  Increased miles/week (group: miles)‡ | RR= 0.57 (0.42 – 0.78)†  RR= 0.58 (0.45 – 0.73)†  RR= 0.90 (0.82 – 0.99)† |
|  |  |  | Knee injuries | Increased miles/week (group: miles)‡  Increased hours/week (group: hours)‡ | RR= 0.90 (0.82 – 0.99)†  RR= 0.49 (0.30 – 0.80)† |
|  |  |  | Foot injuries | Increased hours/week (group: miles)‡  Increased hours/week (group: hours)‡ | RR= 0.31 (0.15 – 0.63)†  RR= 0.21 (0.10– 0.44)† |
|  |  |  | Shin injuries | More intervals§ | RR= 14.89 (0.50 – 147.33)† |
|  | HQ | Van Middelkoop et al., 2008 [42] | Knee injury | M, 100%: Interval (always) | OR= 0.49 (0.26 – 0.93)† |
|  | HQ | Wen et al., 1997 [9] | Shin injuries | Higher proportion of intervals | P= 0.04 |
|  |  |  | Heel injuries | Slower pace | P= 0.034 |
| Surface | HQ | Wen et al., 1997 [9] | Back injuries | Lower percentage training on concrete/asphalt | P= 0.005 |
|  |  |  | Thigh injuries | Lower percentage training on concrete/asphalt | P= 0.011 |
| Distance | HQ | Van Middelkoop et al., 2008 [42] | Incident calf injury | M, 100%: Training distance 0 – 40 km | OR= 0.36 (0.17 – 0.78)† |
|  | HQ | Wen et al., 1997 [9] | Hip injuries | More miles per week | P= 0.035 |
|  |  |  | Hamstring injuries | More miles per week | P= 0.01 |
| Race participation | HQ | Van Middelkoop et al., 2008 [42] | Running injury | M, 100%: Race participation last year: > 7x | OR= 1.55 (1.02- 2.36) |
| Shoe use | HQ | Wen et al., 1997 [9] | Overall injuries | Changing shoes sooner | P= 0.016 |
|  |  |  | Knee injuries | One pair and alternating between 2 pairs | P= 0.037 |
|  | HQ | Wen et al., 1998 [17] | Shin injuries | Higher number of shoes | RR= 6.91 (1.36 – 35.15)† |

† Represents ratios of adjusted OR, HR or RR
§ RRs were calculated dividing the number of injured runners by the total number of runner-weeks accumulated (relative incidence ratios)
‡ RRs were obtained from special subgroups in which information on distances run (miles) and time spent running (hours) was measured
M, Men:: CI, Confidence interval: OR, Odds ratio: ↓ risk, Lower Risk: HR, Hazard ratio: RR, Relative risk: MQ, Methodological quality: HQ, High quality
