# Supplementary material for: Injuries in Runners; A Systematic Review on Risk Factors and Sex Differences
Source: PLoS One. 2015 Feb 23;10(2):e0114937. doi: 10.1371/journal.pone.0114937 (PMC4338213; doi:10.1371/journal.pone.0114937)
Supplement: S6 Table — (DOCX) [file pone.0114937.s009.docx]

**Table S6. Significant health & lifestyle factors related for running injuries**

| **Independent variable** | **MQ** | **Author** | **Injury** | **Specification of independent variable** | **Outcome (95% CI)** |
| --- | --- | --- | --- | --- | --- |
| History of previous injuries | HQ | Bennett et al., 2012 [38] | Exercise-related leg pain (ERLP)  Medial exercise-related leg pain (ERLP) | ERLP history (year)  ERLP history (month)  Medial ERLP history (month) | OR= 18.4 (2.2- 152.7)  OR= 8.5 (2.6- 28.2)  OR= 5.9 (1.4- 25.3) |
|  | HQ | Hirschmüller et al., 2012 [46] | Midportion Achilles tendinopathy (MPT) | Prev. disorders of the Achilles tendon | OR= 3.8 (1.7 – 8.5)† |
|  | HQ | Van Middelkoop et al., 2008 [42] | Running injury | M, 100%: Injury previous 12 months | OR= 2.51 (1.76- 3.56) |
|  |  |  | Knee injury | M, 100%: Injury previous 12 months | OR= 3.67 (1.79 – 7.49)† |
|  |  |  | Calf injury | M, 100%; Incident injury at another localization | OR= 2.57 (1.42 – 4.67)† |
|  | HQ | Wen et al., 1998 [17] | Overall injuries | History of previous injuries§ | RR= 2.02 (1.268 – 3.21)† |
|  |  |  | Shin injuries | History of old shin injuries§ | RR= 7.24 (2.399 – 21.82)† |
| Orthotics | HQ | McKean et al., 2006 [47] | Running injury | Yes (< 40 years)  Yes (≥ 40 years) | P= 0.001  P= 0.001 |
|  | HQ | Wen et al., 1997 [9] | Overall injuries  Foot injuries | Shoe insert use  Shoe insert use | P=0.007  P=0.000 |

† Represents adjusted OR, HR or RR
§ RRs were calculated dividing the number of injured runners by the total number of runner-weeks accumulated (relative incidence ratios)
M, Men: CI, Confidence interval: ERLP, Exercise-related leg pain: OR, Odds ratio: HR, Hazard ratio: RR, Relative risk: MQ, Methodological quality: HQ, High quality
