# Supplementary material for: Injuries in Runners; A Systematic Review on Risk Factors and Sex Differences
Source: PLoS One. 2015 Feb 23;10(2):e0114937. doi: 10.1371/journal.pone.0114937 (PMC4338213; doi:10.1371/journal.pone.0114937)
Supplement: S7 Table — (DOCX) [file pone.0114937.s010.docx]

**Table S7. Risk factors for running injuries with sex ratio**

| **Independent variable** | **Author** | **Injury** | **Specification of independent variable** | **Sex Ratio*** |
| --- | --- | --- | --- | --- |
| Sex | Buist et al., 2010 [22] | Running-related injury (RRI) | M, 42.5% | 0.7 |
|  | McKean et al., 2006 [47] | Running injury | M < 40 yrs. | 0.8† |
| Age | Buist et al., 2010 [10] | Running-related injury (RRI) | M, 32.9%: Increase of age by 10 yrs | 1.4 |
| BMI | Buist et al., 2010 [10] | Running-related injury (RRI) | F, 67.1% | 1.0 |
|  | Buist et al., 2010 [22] | Running-related injury (RRI) | M, 42.5%: 1 kg/m^2^ increase | 0.9 |
| Navicular drop (ND) | Buist et al., 2010 [22] | Running-related injury (RRI) | F, 57.5%: ND (mm) | 0.9 |
| Previous sports activity | Buist et al., 2010 [10] | Running-related injury (RRI) | F, 67.1%: Previously active (non-axial load) | 1.9 |
| Running experience | Buist et al., 2010 [10] | Running-related injury (RRI) | M, 32.9%: Restarting running  F, 67.1%: No previous running experience | 0.7  1.1 |
|  | Macera et al., 1989 [45] | Lower-extremity injury | M, 83.2%: Running experience 0 – 2 yr. | 0.7 |
|  | Macera et al., 1989 [45] | Lower-extremity injury | M, 83.2%: Marathon, during preceding 12 months | 2 |
| Surface | Macera et al., 1989 [45] | Lower-extremity injury | F, 16.8%: Concrete surface | 4.2 |
| Frequency | Macera et al., 1989 [45] | Lower-extremity injury | M, 83.2%: Run 6 or 7 days a week | 0.8 |
| Distance | Macera et al., 1989 [45] | Lower-extremity injury | M, 83.2%: Weekly distance for preceding 3 months; 32- 47.8 km | 0.7 |
|  |  |  | M, 83.2%: Weekly distance for preceding 3 months; 48- 63.8 km | 2.2 |
|  |  |  | M, 83.2%: Weekly distance for preceding 3 months 64.0+ km | 0.4 |
| Shoe use | Taunton et al., 2003 [24] | Overall injury | F, 75.6%: Running shoe age, 4 – 6 months | 4.9† |
| History of previous injuries | Buist et al., 2010 [22] | Running-related injury (RRI) | M, 42.5%: > 3 to ≤ 12 months  M, 42.5%: > 12 months | 0.5  1.0 |
|  | Macera et al., 1989 [45] | Lower-extremity injury | M, 83.2%: Preceding 12 months | 0.7 |

* Sex ratio > 1 represents higher risk for women and sex ratio < 1 represents higher risk for men

† Represents adjusted sex ratio
W, Women; M, men; BMI, Body mass index; ND, Navicular drop
