# Supplementary material for: Injuries in Runners; A Systematic Review on Risk Factors and Sex Differences
Source: PLoS One. 2015 Feb 23;10(2):e0114937. doi: 10.1371/journal.pone.0114937 (PMC4338213; doi:10.1371/journal.pone.0114937)
Supplement: S1 Appendix — (DOCX) [file pone.0114937.s011.docx]

**APPENDIX S1. Search terms PubMed**

(Running[Mesh] OR "Track and Field"[Mesh] OR Runn*[tiab] OR Jogg*[tiab] OR "Track and Field"[tiab])

AND ("Leg Injuries"[Mesh] OR "Hip Injuries"[Mesh:noexp] OR "Knee Injuries"[Mesh:noexp] OR "Ankle Injuries"[Mesh] OR "Foot Injuries"[Mesh] OR ((Injur*[tiab] OR pain[tiab] OR "Bursitis"[Mesh:noexp] OR Bursitis[tiab])

AND (Lower limb[tiab] OR Lower limbs[tiab] OR Lower extremity[tiab] OR lower extremities[tiab] OR leg[tiab] OR legs[tiab] OR hip[tiab] OR hips[tiab] OR knee[tiab] OR knees[tiab] OR ankle[tiab] OR ankles[tiab] OR foot[tiab] OR feet[tiab])) OR "Musculoskeletal Pain"[Mesh] OR "Soft Tissue Injuries"[Mesh] OR "Sprains and Strains"[Mesh:noexp] OR "Tendinopathy"[Mesh] OR Tendinitis[tiab] OR tendinopathy[tiab] OR "Iliotibial Band Syndrome"[Mesh] OR "Patellofemoral Pain Syndrome"[Mesh] OR "Fasciitis, Plantar"[Mesh] OR Iliotibial Band Syndrome[tiab] OR Patella femoral[tiab] OR shin splints[tiab] OR medial tibial stress syndrome[tiab] OR plantar Fasciitis[tiab])

AND ("Risk Factors"[Mesh] OR "etiology"[Subheading:noexp] OR Determinant[tiab] OR determinants[tiab] OR risk[tiab] OR risks[tiab] OR etiology[tiab])

AND ("Cohort Studies"[Mesh] OR Cohort[tiab] OR cohorts[tiab] OR longitudinal[tiab] OR follow-up[tiab] OR followup
